# More than just inflammation: *Ureaplasma* species induce apoptosis in human brain microvascular endothelial cells

**DOI:** 10.1186/s12974-019-1413-8

**Published:** 2019-02-14

**Authors:** Christine Silwedel, Axel Haarmann, Markus Fehrholz, Heike Claus, Christian P. Speer, Kirsten Glaser

**Affiliations:** 1 University Children’s Hospital, University of Wuerzburg, Josef-Schneider-Str. 2, 97080 Wuerzburg, Germany; 2 Department of Neurology, University of Wuerzburg, Josef-Schneider-Str. 11, 97080 Wuerzburg, Germany; 3 Institute for Hygiene and Microbiology, University of Wuerzburg, Josef-Schneider-Str. 2, 97080 Wuerzburg, Germany

**Keywords:** *Ureaplasma urealyticum*, *Ureaplasma parvum*, Neuroinflammation, Meningitis, Caspase, Apoptosis, HBMEC

## Abstract

**Background:**

*Ureaplasma* species (spp.) are commonly regarded as low-virulent commensals but may cause invasive diseases in immunocompromised adults and in neonates, including neonatal meningitis. The interactions of *Ureaplasma* spp. with host defense mechanisms are poorly understood. This study addressed *Ureaplasma*-driven cell death, concentrating on apoptosis as well as inflammatory cell death.

**Methods:**

Human brain microvascular endothelial cells (HBMEC) were exposed to *Ureaplasma* (*U.*) *urealyticum* serovar 8 (Uu8) and *U. parvum* serovar 3 (Up3). Resulting numbers of dead cells as well as mRNA levels and enzyme activity of key agents in programmed cell death were assessed by flow cytometry, RNA sequencing, and qRT-PCR, respectively. xCELLigence data were used for real-time monitoring of changes in cell adhesion properties.

**Results:**

Both *Ureaplasma* isolates induced cell death (*p* < 0.05, vs. broth). Furthermore, *Ureaplasma* spp. enhanced mRNA levels for genes in apoptosis, including caspase 3 (Up3 *p* < 0.05, vs. broth), caspase 7 (*p* < 0.01), and caspase 9 (Up3 *p* < 0.01). Caspase 3 activity was increased upon Uu8 exposure (*p* < 0.01). Vice versa, *Ureaplasma* isolates downregulated mRNA levels for proteins involved in inflammatory cell death, namely caspase 1 (Uu8 *p* < 0.01, Up3 *p* < 0.001), caspase 4 (Uu8 *p* < 0.05, Up3 *p* < 0.01), NOD-like receptor pyrin domain-containing 3 (Uu8 *p* < 0.05), and receptor-interacting protein kinase 3 (*p* < 0.05).

**Conclusions:**

By inducing apoptosis in HBMEC as main constituents of the blood-brain barrier, *Ureaplasma* spp. may provoke barrier breakdown. Simultaneous suppression of inflammatory cell death may additionally attenuate host defense strategies. Ultimate consequence could be invasive and long-term CNS infections by *Ureaplasma* spp.

**Electronic supplementary material:**

The online version of this article (10.1186/s12974-019-1413-8) contains supplementary material, which is available to authorized users.

## Background

Commonly colonizing the adult urogenital tract, the two human *Ureaplasma* species (spp.) *Ureaplasma* (*U.*) *urealyticum* and *U. parvum* are generally regarded as low-virulent commensals [1]. Nonetheless, vertical transmission in pregnancy occurs frequently and appears to be inversely related to maturity [2]. Intra-amniotic *Ureaplasma* infections increase the risk for chorioamnionitis, premature rupture of membranes, and preterm birth [3–5]. Despite ongoing research, however, the implications of a postnatal *Ureaplasma* colonization or infection remain unresolved and appear to be underestimated so far [6]. As well as provoking invasive infections in immunocompromised adults [7–9], *Ureaplasma* spp. may cause pneumonia and sepsis in preterm and term neonates [10, 11]. Furthermore, a growing number of case reports describe *Ureaplasma* spp. as causative agents in neonatal meningitis [12, 13]. Considering typical sequelae of meningitis like cerebral palsy or neurodevelopmental impairment [14, 15], potentially bearing long-term health implications, *Ureaplasma* spp. may have to be regarded of considerable relevance particularly in preterm and term neonates.

Nonetheless, in vitro data addressing the pro-inflammatory capacity of *Ureaplasma* spp. are scarce [16–18]. We recently established a cell culture model of *Ureaplasma* meningitis [19], using human brain microvascular endothelial cells (HBMEC), important constituents of the blood-brain barrier (BBB) and among the first cells to encounter pathogens seeking entry into the central nervous system (CNS) [20]. Having detected *Ureaplasma*-induced responses of atypical chemokine receptor 3, which may ultimately mediate BBB breakdown, we were the first to provide in vitro evidence of *Ureaplasma*-driven neuroinflammation [19].

**Fig. 1:**
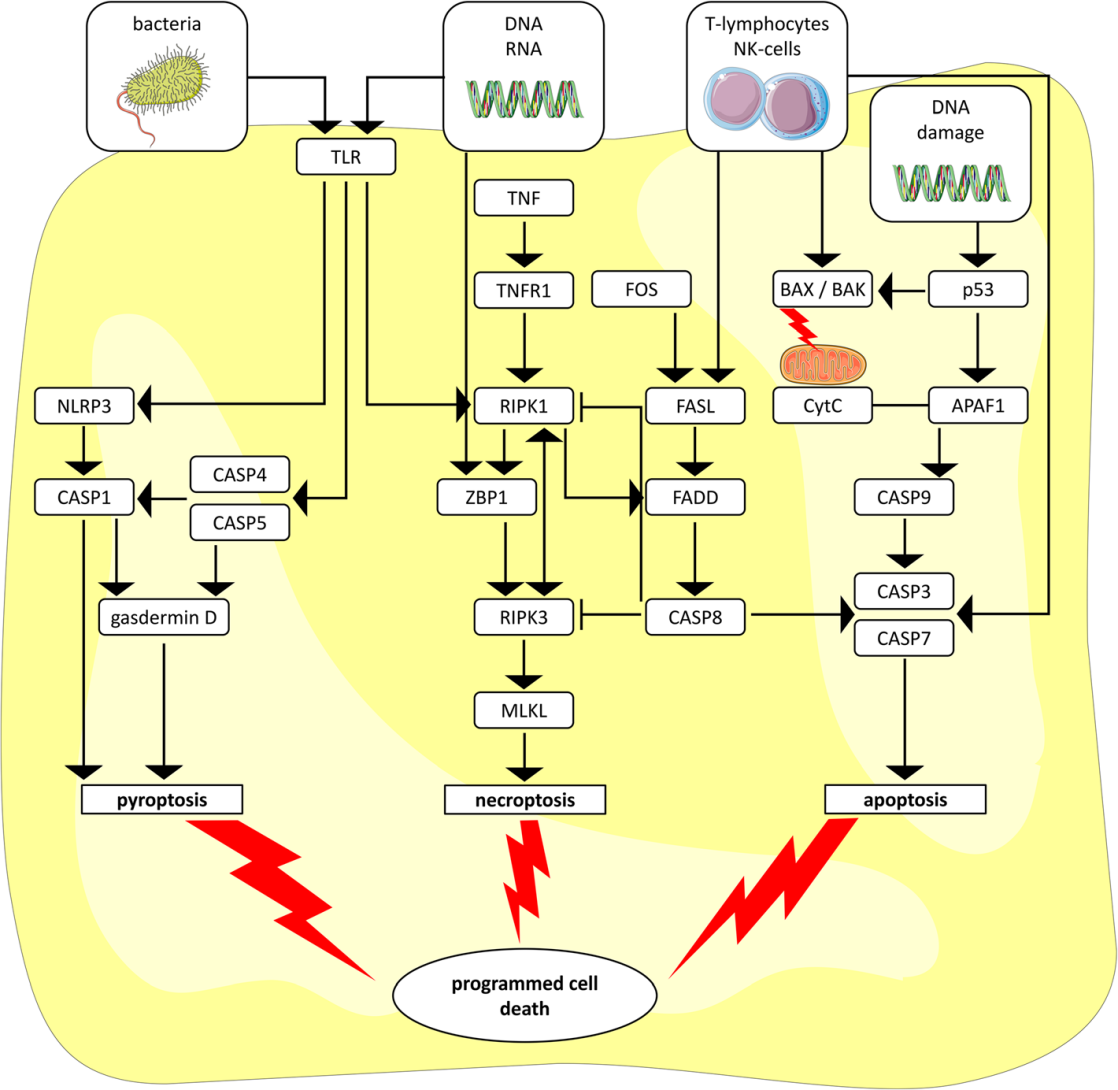
Different forms of programmed cell death and associated cascades. This simplified scheme describes different forms of programmed cell death and the respective key mediators [28–30, 48, 52]. Pyroptosis—bacteria or bacterial components usually employ toll like receptor (TLR) signaling and recruit caspase (CASP) 4 and 5 or CASP1 via inflammasomes, including NOD-like receptor pyrin domain-containing (NLRP) 3. Caspase-driven cleavage of gasdermin D induces membrane rupture and pyroptosis. Necroptosis—DNA or RNA fragments are recognized by Z-DNA binding protein (ZBP) 1 or TLR, which signal via receptor-interacting protein kinase (RIPK) 1. Tumor necrosis factor (TNF) signaling through TNF receptor (TNFR) 1 also involves RIPK1. ZBP1 or RIPK1 activates RIPK3, resulting in recruitment and phosphorylation of mixed lineage kinase-domain-like (MLKL), which ultimately induces pore formation and necroptosis. Apoptosis—lymphocytes, natural killer (NK) cells, or DNA damage (via p53) induce B cell lymphoma (BCL) 2 associated X apoptosis regulator (BAX) or BCL2 homologous antagonist/killer (BAK). BAX and BAK permeabilize the mitochondrial membrane and consecutively released cytochrome C (CytC) binds to apoptotic protease activating factor (APAF) 1, thus forming the apoptosome which activates CASP9. CASP9 activates CASP3 and 7, thereby inducing apoptosis (intrinsic pathway). Inflammatory cells may also directly activate CASP 3 and 7. In an extrinsic apoptosis pathway, first apoptosis signal ligand (FASL) is recruited by immune cells or Fos proto-oncogene (FOS) and activates CASP8 via FAS-associated death domain (FADD). CASP8 then activates CASP 3 and 7. All three processes are closely interacting and are additionally regulated by numerous anti-apoptotic, anti-pyroptotic, and anti-necroptotic proteins not listed here. ├ inhibit / downregulate; ← activate / upregulate / employ. Illustrations: https://smart.servier.com/

Recent studies propose a close association of inflammation and cell death [21, 22]. Induction of cell death in HBMEC is a mechanism some pathogens employ, presumably to gain entrance into the CNS by an impairment of BBB integrity [23–27]. Inflammatory forms of cell death, such as pyroptosis and necroptosis, can be distinguished from the so-called immunologically silent process of apoptosis [28]. As described in Fig. 1, meticulous cascades are involved in either pathway and different forms of cell death are furthermore closely interlinked. Caspases are key mediators among all three of them, with caspases 4 and 5 being involved in inflammatory cell death and caspases 3, 7, and 9 primarily mediating apoptosis [28–30]. Caspases are produced as inactive pro-enzymes which have to be activated by cleavage as a part of the cascades illustrated in Fig. 1 [28, 29].

We used our newly established cell culture model of *Ureaplasma* meningitis to assess induction of cell death with particular focus on caspase levels upon exposure of HBMEC to *Ureaplasma* spp.

## Materials and methods

### Bacterial strains and culture conditions

*U. urealyticum* serovar 8 (Uu8) and *U. parvum* serovar 3 (Up3) were attained from the American Tissue Culture Collection (ATCC; Uu8 ATCC 27618, Up3 ATCC 27815). *Ureaplasma* isolates were cultured in a liquid in-house medium (referred to as “broth”) containing 82% autoclaved pleuropneumonia-like organism medium (Becton, Dickinson & Company, Franklin Lakes, NJ, USA), 10% heat-inactivated horse serum (*v*/*v*), 1% urea (*w*/*v*), and 0.002% phenol red (*w*/*v*) (all from Sigma-Aldrich, St. Louis, CA, USA). After passage through a 0.2-μm filter membrane (Sartorius, Goettingen, Germany), the medium was adjusted to pH 6.5. The ToxinSensor™ Endotoxin Detection System (GenScript, Piscataway, NJ, USA) verified an endotoxin level < 0.06 EU/ml broth. As described previously [17], serial tenfold dilutions of the *Ureaplasma* cultures were incubated for 18–20 h to obtain titers of 1 × 10^9^–1 × 10^10^ color-changing units (CCU)/ml of viable bacteria. Corresponding amounts of *Ureaplasma* DNA were verified and amounted to 5 × 10^7^–6 × 10^8^ copy numbers/ml (Institute of Medical Microbiology and Hospital Hygiene, Duesseldorf, Germany). Simultaneous culture on selective agar plates (medco Diagnostika GmbH, Ottobrunn, Germany) confirmed bacterial viability.

### Cell line and culture conditions

Non-immortalized HBMEC originating from adult human brain cortex (Cell Systems, Kirkland, WA, USA, ACBRI 376) were cultivated in gelatin (Serva Electrophoresis, Heidelberg, Germany) coated T-75 culture flasks (Greiner Bio-One, Frickenhausen, Germany). Cells were propagated in RPMI-1640 medium (Sigma-Aldrich), supplemented with 10% fetal calf serum (FCS) (Thermo Fisher Scientific, Waltham, MA, USA), 10% Nu-Serum (BD Biosciences, San Jose, CA, USA), 2 mM L-glutamine (Thermo Fisher), 1 mM sodium pyruvate (Thermo Fisher), 1% minimum essential medium non-essential amino acids (Thermo Fisher), 5 U/ml heparin (Biochrom, Berlin, Germany), and 0.3% endothelial cell growth supplement (Cell Systems). Cultures were kept in a humid atmosphere at 37 °C with 5% CO_2_. Confluent monolayers were expanded as described previously [19], and experiments were coherently conducted with recently thawed cells at passage 8. Basic endothelial cell attributes of HBMEC (characteristic spindle-shaped growth pattern and expression of the endothelial marker CD31) as well as inducibility of intercellular adhesion molecule 1 had been confirmed in preliminary experiments [19].

### Stimulation assays

For qRT-PCR, RNA sequencing, and flow cytometry, HBMEC were seeded in gelatin-coated 6-well culture plates (Greiner Bio-One) at a density of 2 × 10^5^ cells/well and cultivated for 48 h. Confluent monolayers were washed, and 1 ml fresh growth medium was added per well. As described previously [19], 250 μl broths containing 10^9^–10^10^ CCU *Ureaplasma* were inoculated per milliliter of HBMEC medium. One hundred nanograms per millilter bacterial lipopolysaccharide (LPS, *Escherichia* (*E.*) *coli* serotype 055:B5, Sigma-Aldrich) was added to a subgroup of HBMEC. Cells were stimulated for 4 and 30 h for mRNA analysis and 24 and 48 h for flow cytometry.

For impedance-based real-time monitoring of transendothelial resistance (xCELLigence), HBMEC were transferred to gold electrode-coated plates (Omni Life Science, Bremen, Germany) at a density of 1.25 × 10^4^ cells/well and cultivated in 200 μl growth medium for 48 h. At this point, cells were stimulated in duplicates as described above or left without stimulation.

Inocula and incubation periods had been determined in preliminary experiments [19] analogous to previous approaches [17, 31–34]. Unstimulated HBMEC accounted for negative controls. To adjust for potentially confounding broth effects, cells exposed to *Ureaplasma* isolates were additionally compared to broth control throughout the experiments. In selected experiments, heat-killed *Ureaplasma* isolates (60 °C for 15 min) were additionally used as negative controls.

### RNA extraction and reverse transcriptase PCR (RT-PCR)

Total RNA was extracted using NucleoSpin® RNA Kit (Macherey-Nagel, Dueren, Germany) according to the manufacturer’s instructions. Total RNA was eluted in 60 μl nuclease-free H_2_O (Sigma-Aldrich), quantified using a Qubit® 2.0 Fluorometer (Thermo Fisher), and stored at − 80 °C until reverse transcription. For RT-PCR, 1 μg of total RNA was reverse transcribed using High Capacity cDNA Reverse Transcription Kit (Thermo Fisher) according to the manufacturer’s protocol. First strand cDNA was diluted 1:10 with deionized, nuclease-free H_2_O (Sigma-Aldrich) and stored at − 20 °C until further processing.

### Real time quantitative RT-PCR (qRT-PCR)

For semiquantitative detection of mRNA, cDNA was analyzed in duplicates of 25 μl reaction mixtures containing 12.5 μl iTaq™ Universal SYBR® Green Supermix (Bio-Rad Laboratories, Hercules, CA, USA), 0.5 μl deionized H_2_O, and 1 μl of a 10 μM solution of forward and reverse primer (Sigma-Aldrich), respectively, as indicated in Table 1. PCRs were performed using an Applied Biosystems® 7500 Real-Time PCR System (Thermo Fisher). An initial denaturation at 95 °C for 10 min was followed by 40 cycles of 95 °C for 15 s and 60 °C for 1 min. A melting curve analysis at the end of every run verified single PCR products. Amplification was normalized to the reference gene HPRT1 (hypoxanthine phosphoribosyltransferase 1, Sigma-Aldrich). Mean fold changes in mRNA expression were calculated using the ΔΔC_T_ method by Livak and Schmittgen [35]. Experiments were repeated five times (*n* = 5).

**Table 1:** Primers used for qRT-PCR

Gene symbol	Sequence accession no.	Orientation	Sequence [5′ to 3′]	Amplicon length [bp]
CASP1	NM_033292.3	Forward	AAGTCGGCAGAGATTTATCC	115
		Reverse	ATGTCAACCTCAGCTCCAG	
CASP3	NM_004346.3	Forward	CATTGAGACAGACAGTGG	108
		Reverse	TCGCCAAGAATAATAACCAG	
CASP4	NM_001225.3	Forward	GTTTGACCATCTGCCTCC	126
		Reverse	CGCTGACTCCATATCCCT	
CASP5	NM_004347.3	Forward	CTTTCTGTTCTTCAACACCA	143
		Reverse	ATGATTTCTGTACCTTCCGA	
CASP8	NM_001228.4	Forward	CTGATTCAGAGGAGCAACCC	200
		Reverse	GAATATCATCGCCTCGAGGAC	
CASP9	NM_001229.4	Forward	CCATATCTAGTTTGCCCACAC	183
		Reverse	GAAACAGCATTAGCGACCCT	
HPRT1	NM_000194.2	Forward	CTGGCGTCGTGATTAGTG	121
		Reverse	AGTCCTGTCCATAATTAGTCC	

### RNA sequencing

After the extraction of total RNA (NucleoSpin® RNA Kit, Macherey-Nagel), samples were stored at − 80 °C until analysis. Experiments were repeated three times (*n* = 3). Library preparation was conducted by the Core Unit Systems Medicine, University of Wuerzburg, Germany, using the Illumina TruSeq stranded mRNA Kit (Illumina, San Diego, CA, USA) according to the manufacturer’s instructions, with 700 ng of input RNA and 13 PCR cycles. Thirteen to fourteen pooled libraries were sequenced on a NextSeq 500 (Illumina) with a read length of 75 nucleotides, producing ~ 34–40 million raw reads per library. These were assessed for read quality, amount of duplicates, and presence of adapter sequences employing FastQC 0.11.5 [36]. The Illumina TruSeq adaptors were cleaved using cutadapt (version 1.14) [37], and reads were trimmed keeping a quality drop value below a mean of Q20. Processed sequences were mapped to the human genome using the short read aligner STAR (version 2.5.2b) [38], retrieving genome and annotation files from GENCODE (version 25-March 2016 freeze, GRCh38). The proportion of reads mapped to the human reference genome ranged from 76 to 90% in total for all samples. Sequences aligning to specific genes were quantified with the help of bedtools subcommand intersect (version 2.15.0) [39]. Differentially expressed genes were identified using DESeq2 (version 1.16.1) [40], and differences with a Benjamini-Hochberg corrected *p* value < 0.05 were considered as significant. For the comparison between different groups, reads per kilo base per million mapped reads (RPKM) were calculated employing DGEList and RPKM function from edgeR [41].

### Flow cytometry

Cells were harvested, separated by centrifugation, and stained with Fixable Viability Dye eFluor^TM^ 780 (eBioScience, Thermo Fisher), a dye labelling dead cells. After centrifugation, cells were resuspended in phosphate-buffered saline (PBS, Sigma-Aldrich) and fixed using fixation buffer (BioLegend, San Diego, CA, USA). Centrifugation and permeabilization in permeabilization wash buffer (BioLegend) were followed by staining with antibodies to cleaved caspase 3 (Alexa Fluor 647 conjugated, Cell Signaling Technology, Danvers, MA, USA), caspase 8 (unconjugated, Abcam, Cambridge, UK), and cleaved caspase 9 (PE conjugated, Cell Signaling Technology). Cells were separated by another centrifugation step and were afterwards stained with an Alexa Fluor 405-conjugated secondary antibody (Life Technologies, Thermo Fisher Scientific). After centrifugation, cells were resuspended in PBS containing 1% human serum (Biochrom GmbH) and specimens were read on a FACSCanto™ II flow cytometer (BD Biosciences). A minimum of 10,000 events were acquired and analyzed with FACSDiva v6.1.3 software (BD Biosciences). For viability analysis, all events were included and viability dye positive cells were considered dead. The exact gating strategy for caspase analysis is described in Additional file 1. Experiments were repeated three times (*n* = 3).

### xCELLigence real-time cell monitoring

The xCELLigence system was used to continuously monitor cell adhesion properties. Cells are cultivated in culture plates equipped with microelectrodes, which allow a computer to measure electrical impedance [42]. The xCELLigence software (version 1.2.1.1.002) converts collected data to a cell index. For xCELLigence measurements, HBMEC on gold electrode-coated plates (Omni Life Science) were placed in an ACEA xCELLigence DP system (Omni Life Science) and real-time monitoring of transendothelial resistance was initiated. When forming confluent monolayers, as indicated by a plateau of the cell index, cells were stimulated as described above. Real-time monitoring was continued for 48 h after stimulation. To compare independent experiments, the cell index was normalized to the untreated control and the time point of stimulation. Experiments were repeated five times (*n* = 5).

### Statistical analysis

qRT-PCR, RNA sequencing, and flow cytometry results were analyzed by a one-way ANOVA followed by Tukey’s multiple comparisons test employing Prism® 6 software (GraphPad Software, San Diego, CA, USA). xCELLigence data were assessed by a two-way ANOVA and subsequent Bonferroni test. The significance threshold for *p* values was set at < 0.05. Data are shown as means ± standard deviation (SD).

## Results

### *Ureaplasma*-driven cell death in HBMEC

**Fig. 2:**
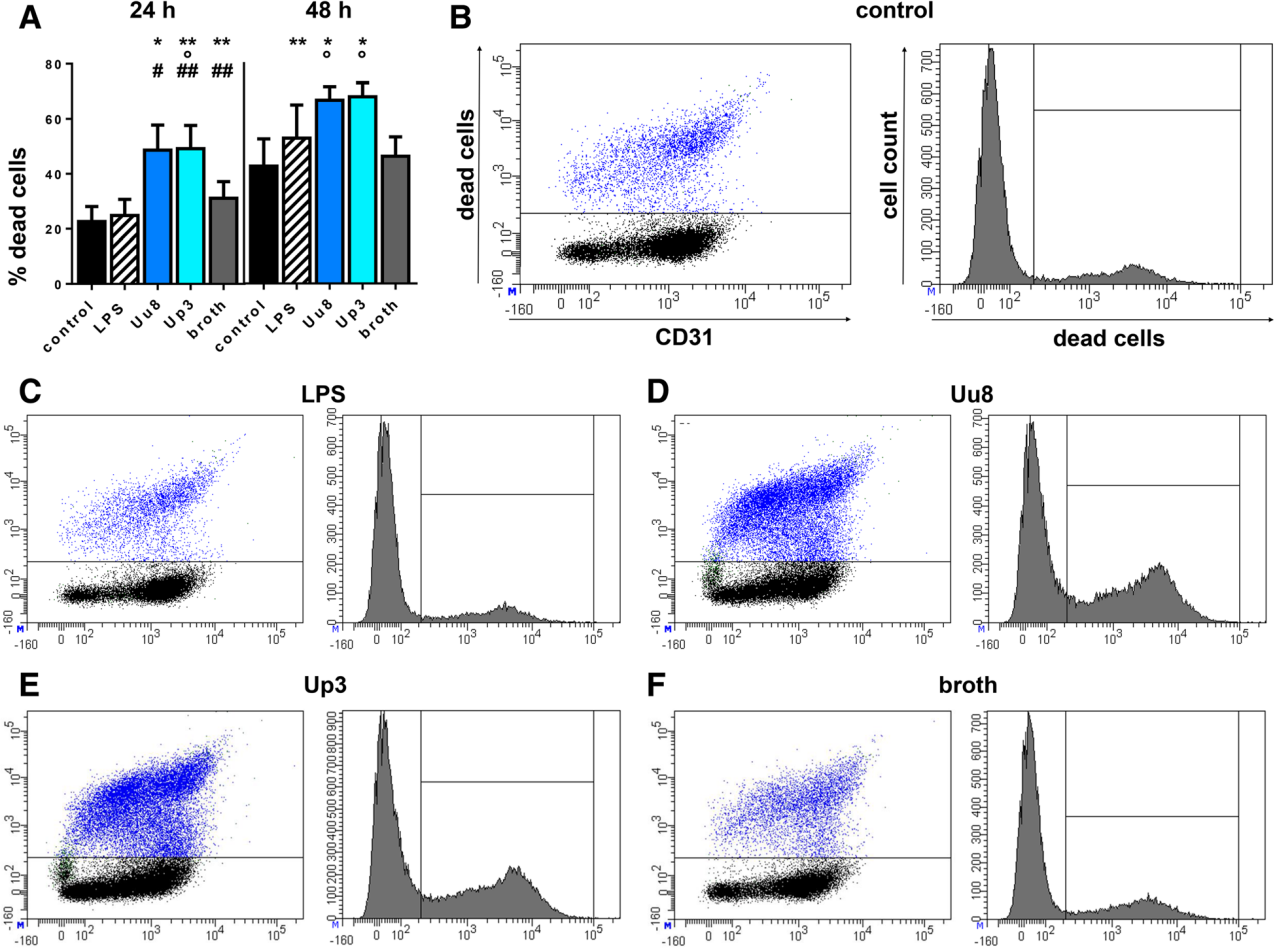
Ureaplasma-induced cell death. Using flow-cytometry and a viability dye labelling dead cells, numbers of non-viable HBMEC were determined for different conditions. **a** Shows the percentage of dead cells upon 24 and 48 h stimulation with *Ureaplasma* and LPS, whereas dot plots for one representative experiment (24 h stimulation) are given in **b**-**f**. Data are presented as means ± SD (*p < 0.05, **p < 0.01 vs. unstimulated control; °*p* < 0.05 vs. broth; ^#^*p* < 0.05, ^##^*p* < 0.01 vs. LPS)

Numbers of viability dye positive cells, considered dead, were determined by flow cytometry (Fig. 2). Even in the absence of any stimulus, control cells underwent cell death over time, but exposure to *Ureaplasma* spp. caused a significant increase in dead cells after 24 h (Uu8 2.15-fold ± 0.4, *p* = 0.0133; Up3 2.17-fold ± 0.4, *p* = 0.0045, vs. control) and 48 h (Uu8 1.59-fold ± 0.1, *p* = 0.0305; Up3 1.59-fold ± 0.1, *p* = 0.0273, vs. control; Fig. 2). In the main, this effect remained significant compared to broth (24 h Uu8 1.56-fold ± 0.3, *p* = 0.0869; Up3 1.58-fold ± 0.3, *p* = 0.0178; 48 h Uu8 1.44-fold ± 0.1, *p* = 0.0375; Up3 1.47-fold ± 0.1, *p* = 0.0281, vs. broth), although broth itself had a mild impact (Fig. 2). LPS did not have any significant effect on cell viability at 24 h, but significantly enhanced numbers of dead cells after 48 h (1.24-fold ± 0.3, *p* = 0.0085, vs. control; Fig. 2).

### *Ureaplasma*-driven apoptosis in HBMEC

**Fig. 3:**
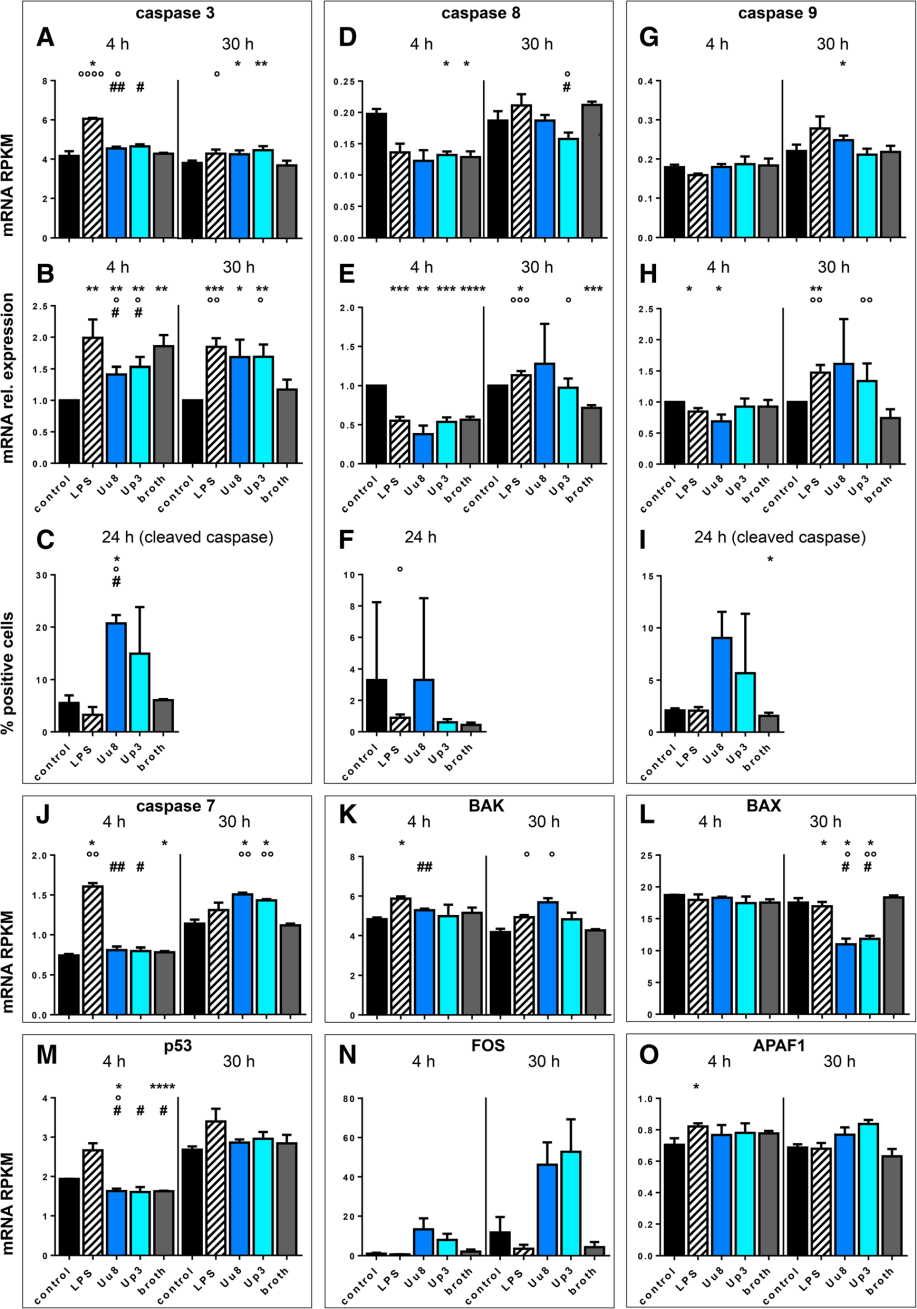
*Ureaplasma*-driven apoptosis in HBMEC. Enzymes and other proteins involved in the apoptotic cascade (Fig. 1) were analyzed upon stimulation of HBMEC for 4 h, 24 h, or 30 h. For caspase 3, mRNA expression was determined via RNA sequencing (**a**) and qRT-PCR (**b**) and enzyme activity (cleaved caspase 3) was assessed via flow cytometry (**c**). For caspase 8, RNA sequencing (**d**) and qRT-PCR (**e**) were used to evaluate mRNA levels and flow cytometry (**f**) was employed to determine protein expression. Caspase 9 mRNA levels were also assessed via RNA sequencing (**g**) and qRT-PCR (**h**), and levels of active caspase 9 were determined using flow cytometry (**i**). RNA sequencing was used to assess mRNA expression of caspase 7 (**j**), BAK (**k**), BAX (**l**), p53 (**m**), FOS (**n**), and APAF1 (**o**). Data are shown as means ± SD (*p < 0.05, **p < 0.01, ***p < 0.001, ****p < 0.0001 vs. unstimulated control; °*p* < 0.05, °°*p* < 0.01, °°°*p* < 0.001, °°°°*p* < 0.0001 vs. broth; ^#^*p* < 0.05, ^##^*p* < 0.01 vs. LPS)

We evaluated some of the key genes in apoptosis described in Fig. 1, correlating mRNA expression obtained by RNA sequencing and qRT-PCR with protein levels or enzyme activity for some of the most important ones (Fig. 3a–i).

RNA sequencing and qRT-PCR revealed enhanced caspase 3 mRNA expression in HBMEC after 30 h of *Ureaplasma* exposure (qRT-PCR Uu8 1.44-fold ± 0.2, *p* = 0.0607; Up3 1.44-fold ± 0.2, *p* = 0.0395, vs. broth; Fig. 3a, b). Levels of cleaved (active) caspase 3 determined via flow cytometry were coherently increased after 24 h of Uu8 exposure (3.41-fold ± 0.3, *p* = 0.0105, vs. broth; Fig. 3c).

Caspase 8 mRNA expression in HBMEC was generally rather low, as deducible from RPKM values no higher than 0.25 (Fig. 3d). No significant effects of *Ureaplasma* stimulation on mRNA or protein levels could be detected compared to control cells and broth control (Fig. 3d–f).

Caspase 9 had an equally low basal expression in HBMEC (Fig. 3g), but RNA sequencing and qRT-PCR revealed a slight mRNA increase after 30 h of *Ureaplasma* exposure, which was significant for Up3 (qRT-PCR 1.80-fold ± 0.4, *p* = 0.0072, vs. broth; Fig. 3g, h). Particularly Uu8 furthermore showed a tendency towards activating caspase 9, although this effect was of borderline significance (5.77-fold ± 1.6, *p* = 0.0939, vs. broth; Fig. 3i).

Using data from RNA sequencing, we analyzed the impact of *Ureaplasma* spp. on mRNA expression of additional apoptosis-related genes (Fig. 3j–o), detecting a significant upregulation of caspase 7 mRNA expression after 30 h (Uu8 1.35-fold ± 0.02, *p* = 0.0083; Up3 1.28-fold ± 0.01, *p* = 0.0034, vs. broth; Fig. 3j). Results furthermore revealed mostly non-significant trends towards higher levels of B cell lymphoma (BCL) 2 homologous antagonist/killer (BAK) (Uu8 1.33-fold ± 0.1, *p* = 0.0357; Up3 1.13-fold ± 0.1, *p* = 0.3089, vs. broth; Fig. 3k), Fos proto-oncogene (FOS) (Uu8 10.6-fold ± 2.6, *p* = 0.1000; Up3 12.1-fold ± 3.8, *p* = 0.1107, vs. broth; Fig. 3n), and apoptotic protease activating factor (APAF) 1 (Uu8 1.21-fold ± 0.1, *p* = 0.3156; Up: 1.33-fold ± 0.04, *p* = 0.1134, vs. broth; Fig. 3o), each after a 30-h stimulation period. Contrarily, *Ureaplasma* spp. significantly decreased BCL2-associated X apoptosis regulator (BAX) mRNA expression in HBMEC (Uu8 0.59-fold ± 0.05, *p* = 0.0180; Up3 0.64-fold ± 0.03, *p* = 0.0048, vs. broth; Fig. 3l), while not exceeding influence on p53 mRNA expression (Fig. 3m). First apoptosis signal (FAS) ligand (FASL) was neither basally expressible nor inducible on mRNA level (data not shown). FAS-associated death domain (FADD) mRNA similarly was not inducible (data not shown).

A shorter stimulation period of 4 h did not provoke any significant *Ureaplasma*-driven mRNA effects if compared to broth and control. *Ureaplasma* isolates did mostly not differ significantly from one another, and broth usually did not have any effect itself.

LPS-induced mRNA responses after a 30-h stimulation period generally resembled *Ureaplasma* effects, and only the downregulation of BAX mRNA was *Ureaplasma* specific (Fig. 3l). Furthermore, the increase in caspase 3 and 9 activity was observable for stimulation with *Ureaplasma* isolates only. Contrarily to the *Ureaplasma* stimulation, however, 4 h exposure of HBMEC to LPS induced significant upregulations of caspase 3 (qRT-PCR 1.99-fold ± 0.3, *p* = 0.0076, vs. control; Fig. 3a, b), caspase 7 (RNA seq 2.16-fold ± 0.1, *p* = 0.0053, vs. control; Fig. 3j), BAK (RNA seq 1.22-fold ± 0.02, *p* = 0.0379, vs. control; Fig. 3k), and APAF1 (RNA seq 1.16-fold ± 0.03, *p* = 0.0384, vs. control; Fig. 3o) mRNA expression.

### *Ureaplasma*-driven pyroptosis in HBMEC

**Fig. 4:**
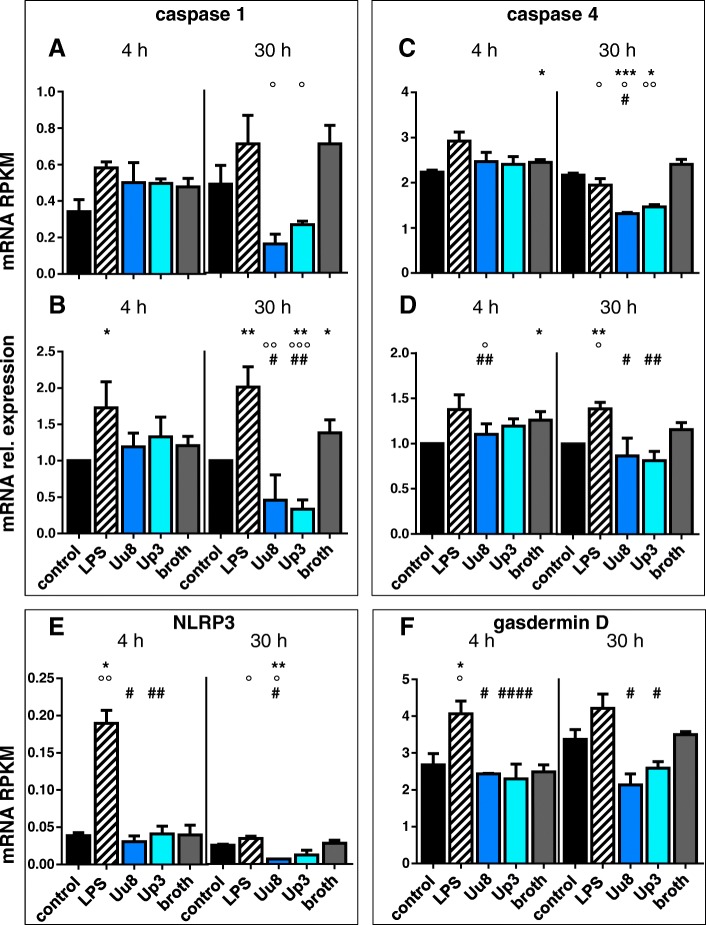
*Ureaplasma*-driven pyroptosis in HBMEC. Key genes in pyroptosis (Fig. 1) were assessed for mRNA responses upon stimulation of HBMEC for 4 h and 30 h. Caspase 1 mRNA expression was determined via RNA sequencing (**a**) and qRT-PCR (**b**). Similarly, RNA sequencing (**c**) and qRT-PCR (**d**) were used to assess caspase 4 mRNA levels. RNA sequencing furthermore determined mRNA expression of NLRP3 (**e**) and gasdermin D (**f**). Data are presented as means ± SD (*p < 0.05, **p < 0.01, ***p < 0.001 vs. unstimulated control; °*p* < 0.05, °°*p* < 0.01, °°°*p* < 0.001 vs. broth; ^#^*p* < 0.05, ^##^*p* < 0.01, ^####^*p* < 0.0001 vs. LPS)

Assessing mRNA levels of some important genes in pyroptosis (described in Fig. 1), we could reveal a significant downregulation of caspase 1 and 4 mRNA expression by *Ureaplasma* isolates. After 30 h of pathogen exposure, RNA sequencing results showed a significant downregulation of caspase 1 mRNA expression (Uu8 0.22-fold ± 0.1, *p* = 0.0154; Up3 0.37-fold ± 0.03, *p* = 0.0367, vs. broth; Fig. 4a) and caspase 4 mRNA expression (Uu8 0.55-fold ± 0.01, *p* = 0.0134; Up3 0.61-fold ± 0.02, *p* = 0.0061, vs. broth; Fig. 4c). Results obtained by qRT-PCR confirmed these findings (Fig. 4b, d). For caspase 5, we observed no basal expression and no induction on mRNA level (data not shown).

We could furthermore observe a significant downregulation of NOD-like receptor pyrin domain-containing 3 (NLRP3) mRNA upon 30 h of Uu8 exposure (0.24-fold ± 0.01, *p* = 0.0325; Fig. 4e), although on low expression levels, as well as a downregulation of borderline significance for gasdermin D mRNA expression after 30 h (Uu8 0.61-fold ± 0.1, *p* = 0.0578; Up3 0.74-fold ± 0.1, *p* = 0.0538, vs. broth; Fig. 4f).

Again, *Ureaplasma* exposure of HBMEC for only 4 h did not result in significant mRNA effects (Fig. 4).

In contradistinction to *Ureaplasma* isolates, stimulation of HBMEC with LPS for 4 as well as 30 h enhanced mRNA levels of all given pyroptosis genes. We observed significant LPS-driven differences for caspase 1 (4 h qRT-PCR 1.73-fold ± 0.4, *p* = 0.0469; 30 h qRT-PCR 2.0-fold ± 0.3, *p* = 0.0059, vs. control; Fig. 4a, b), caspase 4 (30 h qRT-PCR 1.39-fold ± 0.1, *p* = 0.0072, vs. control; Fig. 4c, d), NLRP3 (4 h RNA seq 4.88-fold ± 0.5, *p* = 0.0144, vs. control; Fig. 4e), and gasdermin D (4 h RNA seq 1.52-fold ± 0.1, *p* = 0.0266, vs. control; Fig. 4f).

### *Ureaplasma*-driven necroptosis in HBMEC

**Fig. 5:**
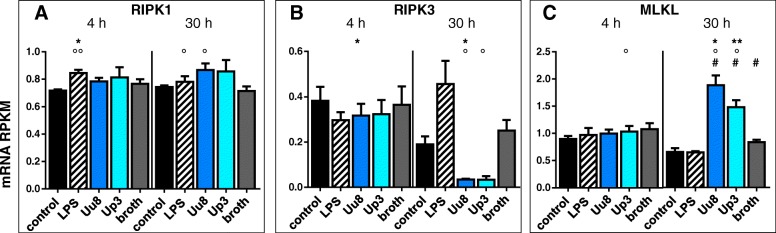
*Ureaplasma*-driven necroptosis in HBMEC. RNA sequencing data were used to assess stimulation-induced mRNA responses for RIPK1 (**a**), RIPK3 (**b**), and MLKL (**c**) as important factors in necroptosis. Data are shown as means ± SD (*p < 0.05 vs. unstimulated control; °*p* < 0.05, °°*p* < 0.01 vs. broth)

Stimulation of HBMEC with *Ureaplasma* isolates for 30 h resulted in a significant downregulation of receptor-interacting protein kinase (RIPK) 3 mRNA levels (Uu8 0.14-fold ± 0.02, *p* = 0.0463; Up3 0.13-fold ± 0.06, *p* = 0.0493, vs. broth; Fig. 5b). For RIPK1, we observed a mild mRNA upregulation upon *Ureaplasma* exposure, which was, however, not significant compared to control cells (Fig. 5a). Mixed lineage kinase-domain-like (MLKL) mRNA levels were significantly enhanced after 30 h of *Ureaplasma* exposure (Uu8 2.26-fold ± 0.2, *p* = 0.0172; Up3 1.77-fold ± 0.2, *p* = 0.0283, vs. broth; Fig. 5c). Z-DNA binding protein (ZBP) 1 was neither basally expressible nor inducible in HBMEC (data not shown).

LPS stimulation significantly enhanced RIPK1 mRNA levels after 4 h (1.18-fold ± 0.03, *p* = 0.0153, vs. control; Fig. 5a) and evoked a trend towards increased mRNA expression of RIPK3 after 30 h (2.4-fold ± 0.5, *p* = 0.1444, vs. control; Fig. 5b), while not influencing MLKL mRNA expression.

Effects of stimulation on FASL, FADD, CASP8, and FOS, involved in both necroptosis and apoptosis, are described above (“*Ureaplasma*-driven apoptosis in HBMEC”).

### *Ureaplasma*-driven impairment of HBMEC barrier properties

**Fig. 6:**
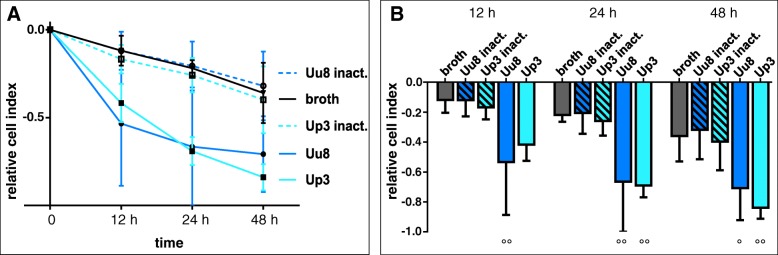
*Ureaplasma*-driven impairment of HBMEC barrier properties. xCELLigence measurements were employed to assess long-term changes of endothelial adhesion upon stimulation, with the relative cell index depicting variances in relation to unstimulated control cells. **a** relative cell index for stimulation with Uu8 and Up3 vs. exposure to broth or heat-inactivated isolates over a period of 48 h. **b** comparison between different conditions. Data are presented as means ± SD (°*p* < 0.05, °°*p* < 0.01, vs. broth)

xCELLigence data were used to continuously monitor changes in cell adhesion properties evoked by stimulation with *Ureaplasma* spp. As illustrated in Fig. 6, both *Ureaplasma* isolates progressively reduced endothelial barrier properties over time. Compared to broth, *Ureaplasma* spp. significantly reduced the relative cell index after 12 h (Uu8 4.48-fold ± 3.0, *p* = 0.0097, vs. broth), 24 h (Uu8 3.04-fold ± 1.5, *p* = 0.0048; Up3 3.15-fold ± 0.4, *p* = 0.0060, vs. broth), and 48 h (Uu8 1.97-fold ± 0.6, *p* = 0.0388; Up3 2.33-fold ± 0.2, *p* = 0.0051, vs. broth). Broth itself had some negative impact on cell adhesion, which increased with the duration of the experiment, whereas heat-inactivated *Ureaplasma* isolates yielded no different effects than broth.

## Discussion

This study is the first to provide in vitro insights into strategies *Ureaplasma* spp. use to differentially interfere with programmed cell death in HBMEC. Programmed cell death is an immune defense mechanism host cells employ to eliminate particularly intracellular pathogens [28]. On the less favorable side, however, cell death may also benefit the pathogen by reduction of immune cell numbers or impairment of physiological barriers. For the first time, we could provide evidence for *Ureaplasma*-driven induction of apoptosis and simultaneous suppression of inflammatory forms of cell death in HBMEC.

Whereas caspases are important contributors to all forms of programmed cell death (Fig. 1), caspases 3, 7, and 9 seem to be primarily involved in apoptosis [29]. Our data demonstrate an *Ureaplasma*-driven upregulation of caspase 3 and caspase 9 mRNA after 30 h as well as an increase in enzyme activity (Fig. 3a–c, g–i). Similarly, caspase 7 mRNA expression was enhanced after 30 h of *Ureaplasma* exposure (Fig. 3j) and we could furthermore observe a trend towards elevated mRNA levels for BAK, FOS, and APAF1 (Fig. 3k, n, o) as additional pro-apoptotic proteins. These results suggest an ability of *Ureaplasma* spp. to induce apoptosis. Acknowledging the role of the individual proteins in the apoptotic cascade (Fig. 1), *Ureaplasma* spp. appear to primarily activate the so-called intrinsic apoptotic pathway in HBMEC. *Ureaplasma*-driven upregulation of FOS mRNA furthermore indicates an activation of the extrinsic pathway as well, although FASL, FADD, and caspase 8 as downstream effectors do not seem to be involved on mRNA levels. This *Ureaplasma*-driven induction of apoptosis is reflected in a significant increase in cell death upon 24 and 48 h exposure of HBMEC to *Ureaplasma* spp. (Fig. 2).

Whereas induction of apoptosis in HBMEC has been described for other pathogens or bacterial components, including listeriolysin O as well as *E. coli* shiga toxin and hemolysin [25–27], we are the first to provide evidence for *Ureaplasma*-driven apoptosis in HBMEC. As main components of the BBB, apoptosis of HBMEC may consecutively result in BBB breakdown. We could indeed verify *Ureaplasma*-driven impairment of HBMEC barrier properties by continuously monitoring cell adhesion properties using the xCELLigence technique. Of note, other authors using the xCELLigence system described loss of cell adhesion in apoptotic but not in necrotic cell death [43].

Recently having demonstrated the upregulation of atypical chemokine receptor 3 in HBMEC by *Ureaplasma* spp., potentially causing BBB impairment [19], induction of apoptosis appears to be yet another method these pathogens employ to disturb the protective CNS barrier. Numerous neuroinflammatory diseases are facilitated by a compromised BBB integrity [20, 44]. *Ureaplasma*-driven BBB impairment may (1) allow *Ureaplasma* entry into the CNS, (2) predispose for invasive CNS infections with other pathogens, and (3) facilitate inflammatory cell influx into the CNS, causing chronic intracerebral inflammation. In line with this, *Ureaplasma* spp. have not only been identified as causative pathogens in neonatal meningitis, but have also been associated with cerebral palsy or intraventricular hemorrhage in preterm infants [6, 12, 13, 45, 46]. Of note, white matter injury, in particular, has also been associated with endothelial cell apoptosis and BBB breakdown [47]. *Ureaplasma*-driven induction of apoptosis and BBB impairment could thus be the common pathological feature in several neuroinflammatory diseases of prematurity.

We could recently demonstrate *Ureaplasma*-induced downregulation of apoptotic caspase mRNA in pulmonary epithelial cells, but a converse upregulation of apoptotic caspase protein or activity in pulmonary microvascular endothelial cells (submitted manuscript). In line with the findings presented in this study, *Ureaplasma* spp. may thus specifically induce cell death in microvascular endothelial cells, facilitating their own invasion from the blood stream into the respective tissue. Once there, pathogens may then reciprocally inhibit apoptosis to prevent eradication and establish chronic infections with corresponding long-term sequelae.

In line with this theory, *Ureaplasma* spp. appear to suppress pyroptosis and potentially necroptosis in HBMEC. For several involved genes in both pathways, we could observe a downregulation of mRNA levels after 30 h of *Ureaplasma* exposure. This includes caspases 1 and 4, NLRP3, and gasdermin D for pyroptosis (Fig. 4) as well as the necroptotic kinase RIPK3 (Fig. 5b). Only for MLKL, the executioner of necroptosis, *Ureaplasma* exposure of HBMEC resulted in a distinct upregulation of mRNA expression (Fig. 5c). However, regardless of its mRNA amount, MLKL needs to be phosphorylated by RIPK3 to fulfill its role in necroptosis [48]. Other authors demonstrated that a lack of RIPK3 mRNA resulted in absent MLKL phosphorylation and inhibition of necroptosis [49]. We speculate that, by suppressing key participants in inflammatory cell death, *Ureaplasma* spp. may concurrently attenuate several important immune defense mechanisms. First of all, pyroptosis and necroptosis are considered effective mechanisms to eliminate intracellular pathogens [24, 29, 50–52]. Furthermore, particularly caspase 4 has been shown to trigger not only pyroptosis, but also the production of the pro-inflammatory cytokine interleukin (IL)-1β [53, 54], thus interlinking cell death and inflammation. Therefore, *Ureaplasma*-driven downregulation of caspase 4 may additionally suppress secretion of IL-1β and thus attenuate important pro-inflammatory cytokine cascades.

The exact mechanisms of *Ureaplasma*-driven modulation of programmed cell death are yet to be determined. Evaluating virulence factors of *Ureaplasma* spp. in the context of well-known factors initiating or inhibiting apoptosis, pyroptosis, and necroptosis (Fig. 1), it seems reasonable to speculate an involvement of TLR signaling, *Ureaplasma*-driven TNF-α protein release (recently demonstrated for human monocytes [17]), and potentially cell invasion and ammonia production by *Ureaplasma* spp.

Interestingly, LPS, a component of bacterial membranes used to mimic bacterial infections, induced effects widely differing from *Ureaplasma*-evoked responses. Following LPS exposure of HBMEC, we could observe an early upregulation of apoptotic cascade mRNA levels after only 4 h (Fig. 3), whereas *Ureaplasma* spp. generally evoked responses solely over longer stimulation periods. This fierce and immediate LPS effect is reflected in the fulminant clinical course of a typical bacterial meningitis, whereas the ramifications of an infection with *Ureaplasma* spp. may be confined in the beginning, but increase over time. Studies describe cases of chronic *Ureaplasma* meningitis with a history of several months [12, 13]. Furthermore, unlike the downregulation we could observe upon *Ureaplasma* exposure, LPS enhanced caspase 1, caspase 4, NLRP3, and gasdermin D as well as RIPK1 mRNA expression in HBMEC (Figs. 4 and 5), thus apparently triggering pyroptosis and necroptosis. Nonetheless, LPS did not increase the overall number of dead HBMEC upon a 24-h stimulation period and only began to do so after 48 h (Fig. 2), with effects less pronounced than the ones evoked by *Ureaplasma* spp. It is therefore likely that LPS simultaneously enhances protective factors which confine cell death. *Ureaplasma* spp., on the other hand, appear to disturb the pro- and anti-apoptotic equilibrium and cause extensive cell damage. In line with this speculation, we were able to demonstrate an increase in mRNA and protein expression of anti-inflammatory IL-10 induced by LPS, but not by *Ureaplasma* isolates in primary human monocytes [17]. IL-10 has been shown protective against apoptosis in HBMEC [55].

Simultaneous strength and limitation of this study is the use of a non-immortalized cell line. Primary cell lines closely resemble in vivo cells, but also have a limited in vitro life span. This is reflected in a relevant number of dead control cells particularly after 48 h (96 h after seeding) and in a decrease in transendothelial resistance in control cells over time. Aggravating factors may be lack of substrate and lack of space after confluency.

This study provides a thorough assessment of complex cascades involved in different forms of programmed cell death, correlating the basic cellular processes with actual ramifications on cell viability and, by monitoring barrier properties, even physiological functioning. We did, however, concentrate primarily on mRNA levels and can provide protein or enzyme activity data only for a few of many participants. Under in vivo conditions, activation processes and complex interactions are involved in the regulation of caspases and other enzymes [30] and in vitro settings cannot be fully representative. Further studies functionally analyzing the individual cascades, assessing associated signaling pathways, and including additional apoptosis assays are therefore essential to improve our understanding of *Ureaplasma*-driven cell death.

## Conclusions

Due to their high prevalence, difficult cultural detection, and contradictory clinical data, the relevance of *Ureaplasma* spp. is still subject of controversial discussion. Having recently demonstrated a pro-inflammatory capacity of *Ureaplasma* spp. in HBMEC, the present study reveals that the injurious effects of *Ureaplasma* spp. go far beyond an ability to “merely” cause inflammation. Our in vitro findings suggest *Ureaplasma*-driven apoptosis in HBMEC, which, in vivo, may ultimately cause BBB breakdown with resulting CNS inflammation. A potential additional suppression of inflammatory forms of cell death by *Ureaplasma* spp. may further impair host immune defense mechanisms and ultimately facilitate long-term cerebral infections. We appear to be confronted with a meticulously acting pathogen of still underestimated clinical relevance.

## Additional file

Additional file 1: Gating strategy for analysis of caspase flow cytometry results. Events were gated via forward and side scatter, and a SSC-height versus FSC-width dot plot was used to exclude doublets. Single cells were then gated for viability dye negative, caspase positive cells and numbers were depicted in the respective figures (Fig. 3c, f, i). CASP: caspase. (TIF 2881 kb)

## Abbreviations

APAF: Apoptotic protease activating factor
ATCC: American Tissue Culture Collection
BAK: BCL2 homologous antagonist/killer
BAX: BCL2-associated X apoptosis regulator
BBB: Blood-brain barrier
BCL: B cell lymphoma
CCU: Color-changing units
CNS: Central nervous system
CytC: Cytochrome C
E: Escherichia
FADD: FAS-associated death domain
FAS: First apoptosis signal
FASL: FAS ligand
FCS: Fetal calf serum
FOS: Fos proto-oncogene
HBMEC: Human brain microvascular endothelial cells
HPRT1: Hypoxanthine phosphoribosyltransferase 1
IL: Interleukin
LPS: Lipopolysaccharide
MLKL: Mixed lineage kinase-domain-like
NLRP3: NOD-like receptor pyrin domain-containing
PBS: Phosphate-buffered saline
qRT-PCR: Real-time quantitative reverse transcriptase polymerase chain reaction
RIPK: Receptor-interacting protein kinase
RPKM: Reads per kilo base per million mapped reads
SD: Standard deviation
spp.: Species
TLR: Toll-like receptor
TNF: Tumor necrosis factor
TNFR: TNF receptor
U: *Ureaplasma*
Up3: *Ureaplasma parvum* serovar 3
Uu8: *Ureaplasma urealyticum* serovar 8
ZBP: Z-DNA binding protein
